# Mannosylated neutrophil vesicles targeting macrophages alleviate liver inflammation by delivering CRISPR/Cas9 RNPs

**DOI:** 10.7150/thno.107791

**Published:** 2025-05-08

**Authors:** Dongqing Wu, Hang Shu, Mengmeng Zhang, Xiaoli Wei, Jingjing Ji, Haiyuan Shen, Hejiao Zhang, Linxi Xie, Liangliang Zhou, Lei Yang, Jiali Jiang, Chen Chen, Shanfei Tian, Xinru Zhang, Xu Long, Xiaoyan He, Hua Wang

**Affiliations:** 1 Department of Oncology, the First Affiliated Hospital of Anhui Medical University, Hefei 230000, Anhui, China; 2 School of Pharmaceutical Sciences, Anhui Medical University, Hefei 230032, China.; 3 Department of Gastroenterology, the First Affiliated Hospital of Anhui Medical University, Hefei 230000, Anhui, China; 4 School of Basic Medical Science, Anhui Medical University, Hefei 230000, Anhui, China; 5 School of Life Sciences, Anhui Medical University, Hefei 230000, Anhui, China; 6 School of Pharmacy, Inflammation and Immune-Mediated Diseases Laboratory of Anhui Province, Anhui Medical University, Hefei 230000, Anhui, China; 7 Innovation and Entrepreneurship Laboratory for College Students, Anhui Medical University, Hefei 230000, Anhui, China

**Keywords:** mannosylated neutrophil, hepatic macrophages, NLRP3 gene, inflammatory liver diseases

## Abstract

**Background:** Inflammation is a key driver of various liver diseases. NLRP3 inflammasome in hepatic macrophages is a key mediator of inflammation and has emerged as a promising target. Genome editing presents a powerful approach to modulate inflammation by directly disrupting genes such as NLRP3 directly. However, efficient and cell-specific delivery of CRISPR/Cas9 ribonucleoproteins (RNPs) remains challenging.

**Methods:** We developed a novel delivery system by encapsulating CRISPR/Cas9 RNPs within mannosylated neutrophil membranes vesicles (Cas9/gNLRP3@M-N) to enhance targeting hepatic macrophages.

**Results:** Cas9/gNLRP3@M-N selectively accumulated in hepatic macrophages, effectively disrupted the NLRP3 gene, attenuated inflammation in acute fulminant hepatitis, and improved disease outcomes in chronic steatohepatitis model.

**Conclusions:** Cas9/gNLRP3@M-N represents a promising targeted gene-editing approach for the treatment of inflammatory liver diseases.

## Introduction

Liver disease is a major global health burden, accounting for over two million deaths annually and representing approximately 4% of worldwide mortality 1. Despite its high prevalence, effective treatment for many liver conditions remains limited. Inflammatory liver diseases, including fulminant hepatitis (FH) and metabolic dysfunction-associated steatohepatitis (MASH), are among the leading causes of irreversible liver diseases and progressive fibrosis 2. FH is a life-threatening multi-system syndrome triggered by drugs or infections and remains associated with high mortality. Liver transplantation continues to be required for FH despite improvements in clinical care over the past two decades. Currently, no curative treatments exist for FH 3. Similarly, MASH, previously known as nonalcoholic steatohepatitis, is now recognised as a progressive form of metabolic dysfunction-associated steatotic liver disease (MASLD), formerly termed nonalcoholic fatty liver disease 4. As the most common chronic liver disease globally, MASH can progress to advanced fibrosis, cirrhosis, and hepatocellular carcinoma, posing a significant global health challenge 5.

Hepatic macrophages are the most abundant innate immune cells in the liver and play a central role in orchestrating localized inflammation 6. When dysregulated, these macrophages can trigger uncontrolled inflammatory responses, disrupting homeostasis and repair mechanisms, and contributing to liver disease progression, including FH and MASH. The NLRP3 inflammasome, a well-characterized pro-inflammatory protein complex, is particularly active in macrophages and is a key driver of pathological macrophage activation 7. Consequently, targeting macrophage/NLRP3 activation has been proposed as a therapeutic strategy for inflammatory liver diseases. However, to date, no drugs that directly target the NLRP3 inflammasome have reached the clinic 8. Current NLRP3-associated treatments primarily target downstream cytokines such as IL-1β 9. Thus, the development of direct NLRP3 inhibitors is of great clinical interest.

The clustered regularly interspaced short palindromic repeat (CRISPR)/CRISPR-associated nuclease 9 (Cas9) system offers a powerful and precise approach for site-specific genome editing 10. With several CRISPR-based therapies for monogenic disorders already in clinical trials 11, this technology holds promise for treating previously intractable diseases, including inflammatory diseases driven by aberrant NLRP3 activity 12. However, the efficacy of CRISPR/Cas9-based treatments hinges on the efficient and specific delivery to target cells. Delivery methods include plasmid DNA, mRNA, or preassembled Cas9/gRNA ribonucleoproteins (RNPs) 13. Among these, direct delivery of Cas9/gRNA RNPs offers several advantages: rapid editing, minimized off-target effects, and reduced immunogenicity 14. Yet, the large molecular size of Cas9 and the susceptibility of Cas9/gRNA RNPs to degradation remain key barriers for efficient delivery and broad application. Current non-viral delivery strategies - including physical and chemical (e.g., electroporation, polymer nanoparticles, lipid nanoparticles) - have both strengths and limitations 15-17. Achieving safe, efficient, and cell-specific delivery of Cas9/gRNA RNPs *in vivo* remains a critical challenge in gene therapy 18.

Cell membrane coating has emerged as an attractive platform technology for therapeutic delivery 19. By coating nanoparticles with cell membranes, these particles can adopt the biocompatibility and functional characteristics of the source cells. Advances in cell membrane-mimicking nanoparticles for drug delivery have spurred interest in using them as delivery vehicles for Cas9/gRNA RNPs, potentially overcoming several limitations in gene therapy.

**Figure 1:**
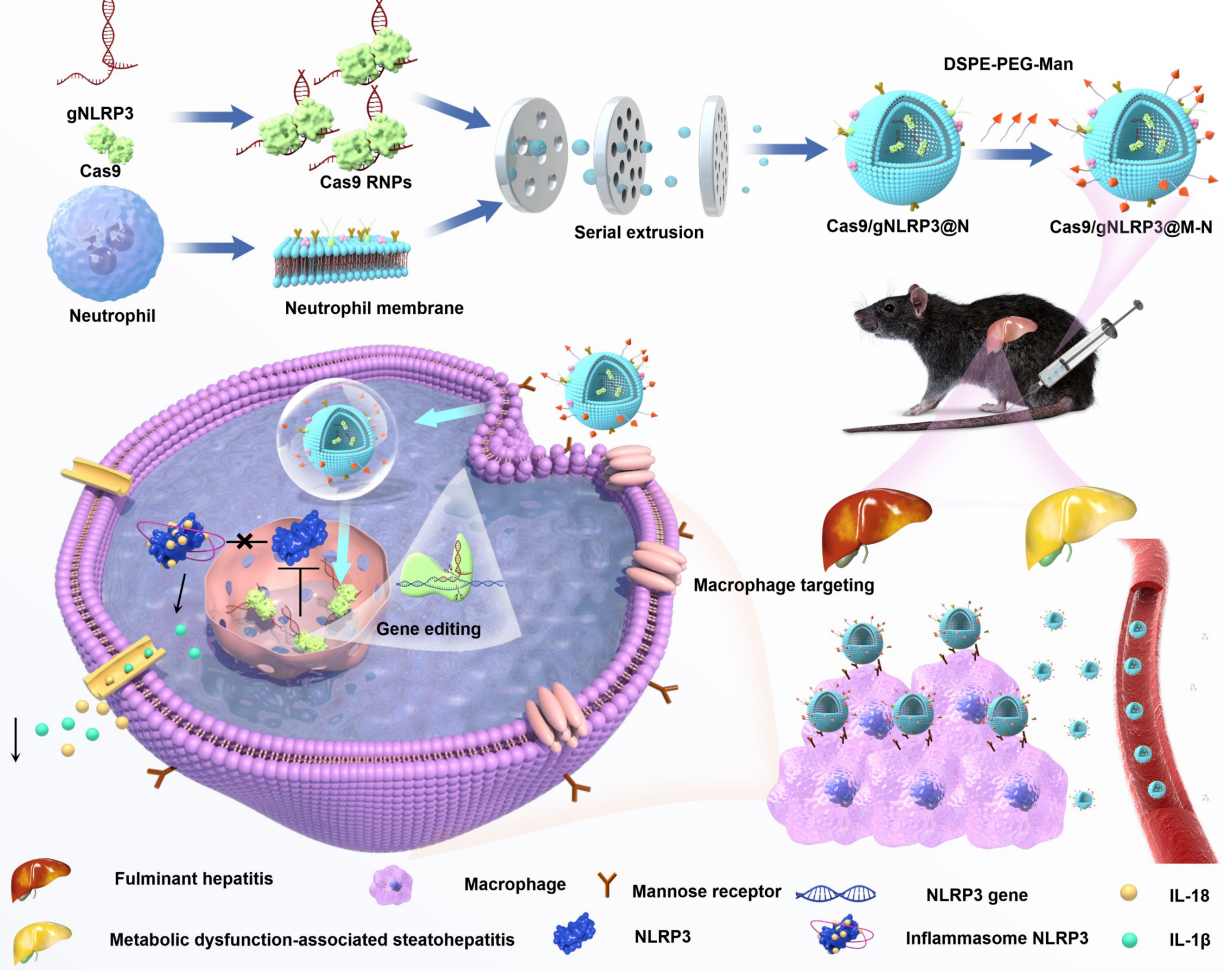
Schematic illustration of the fabrication, delivery, and intracellular fate of Cas9/gNLRP3@M-N.

In this study, we encapsulated Cas9 and a guide RNA within neutrophil membranes to create Cas9/gNLRP3@N. We then enhanced macrophage targeting by integrating DSPE-PEG-Man ligands into the lipid bilayer, generating Cas9/gNLRP3@M-N. Under inflammatory conditions, Cas9/gNLRP3@M-N demonstrated dual targeting to the liver and macrophages. In hepatic macrophages, Cas9/gNLRP3@M-N effectively suppressed NLRP3 *in vivo* and significantly reduced inflammation in both LPS/D-GalN-induced FH and two diet-induced MASH models (Figure 1). Our findings present a promising strategy for CRISPR/Cas9 delivery to macrophages and offer a novel therapeutic approach for multiple inflammatory liver diseases.

## Results

### Preparation and characterization of the Cas9/gNLRP3@M-N

**Figure 2:**
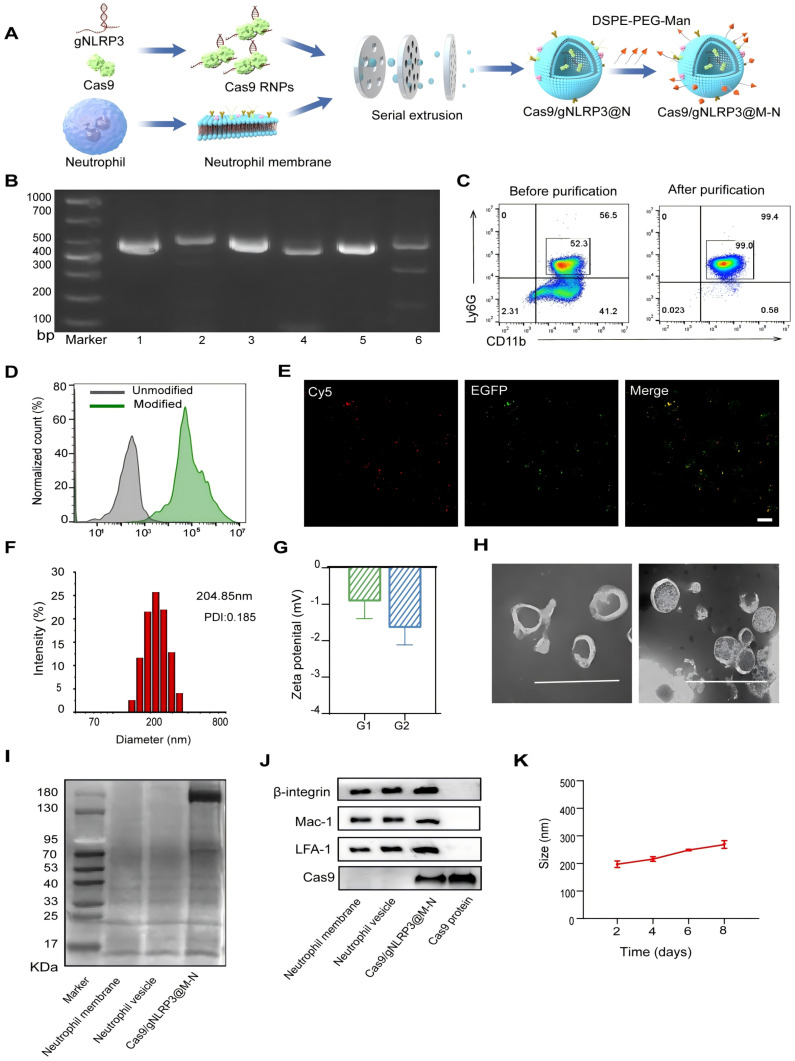
Preparation and characterization of Cas9/gNLRP3@M-N. A) Schematic of the preparation of Cas9/gNLRP3@M-N. B) *In vitro* cleavage of target DNA with RNP. 1: DNA substrate1, 2: DNA substrate1 + Cas9/gRNA-1, 3: DNA substrate2, 4: DNA substrate2 + Cas9/gRNA-2, 5: DNA substrate3, 6: DNA substrate3 + Cas9/gRNA-3. C) Flow cytometry analysis of mouse bone marrow neutrophils (gate CD45^+^). D) Flow cytometry analysis of unmodified and modified empty neutrophil vesicles with DSPE-PEG-Cy5. E) Colocalization of DSPE-PEG-Man and EGFP-labeled Cas9 upon intracellular uptake by RAW246.7 cells. Scale bars: 20 μm. F) The hydrodynamic diameter of Cas9/gNLRP3@M-N was analyzed by dynamic light scattering (DLS). G) Zeta potential of the neutrophil vesicle(G1), and Cas9/gNLRP3@M-N(G2). H) The morphology of Cas9/gNLRP3@M-N was analyzed by transmission electron microscopy (TEM). Scale: 500 nm. I) SDS-PAGE protein analysis of neutrophil membrane, neutrophil vesicle, and Cas9/gNLRP3@M-N. J) Characteristic protein expression from the Cas9/gNLRP3@M-N. K) Stability of Cas9/gNLRP3@M-N over time in phosphate buffered saline (PBS) for 1 week (stored at 4 ℃ in the dark).

Cas9/gNLRP3@M-N was prepared in four main steps: (i) synthesis of Cas9 RNPs, (ii) purification of neutrophil membranes, (iii) fabrication of neutrophil vesicle particles, and (iv) insertion of mannose ligands into the vesicles (Figure 2A). First, Cas9 proteins with nuclear localization signals (NLS) were purified from an E. coli expression system and demonstrated a robust cleavage activity (Figure S1). gRNAs were synthesized via *in vitro* transcription, and Cas9 was assembled with gRNAs at a 1:3 molar ratio, following previously published guidelines 20-22. Among three different gRNAs targeting NLRP3, Cas9/gNLRP3-3 showed the highest cleavage efficiency and was selected for further experiments (Figure 2B).

Neutrophil membranes were purified from bone marrow-derived mouse neutrophils (Figure S2, 2C). Cas9/gNLRP3 RNPs were then co-extruded with neutrophil vesicles to form Cas9/gNLRP3@N. To improve macrophage targeting, mannose (Man) ligands were incorporated due to the high expression of mannose receptors on macrophages 23. Mannose was conjugated to DSPE-PEG-NH_2_ to create mannosylated lipid-ligand DSPE-PEG-Man (Figure S3A), confirmed by ^1^H NMR analysis (Figure S3B). This mannose-modified ligand was integrated onto the surface of Cas9/gNLRP3@N to produce Cas9/gNLRP3@M-N with macrophage-targeting capabilities.

Fluorescence microscopy confirmed successful coating, with neutrophil vesicles co-incubated with DSPE-PEG-Cy5 fully acquiring Cy5 fluorescence (Figure 2D, S4A). Additionally, GFP-labeled NLS-Cas9 co-localized with DSPE-PEG-Cy5 (Figure 2E), verifying successful surface modification. Dynamic light scattering (DLS) measurements revealed that the hydrodynamic diameter of Cas9/gNLRP3@M-N increased by approximately 42 nm compared to empty neutrophil vesicles, with a low polydispersity index (PDI), indicating high uniformity (Figure S4B, 2F). Zeta potential of Cas9/gNLRP3@M-N was similar to that of empty vesicles (Figure 2G), and both exhibited typical ≈200 nm saucer-shaped nanovesicles, indicating consistent membrane coating (Figure 2H). Western blot analysis confirmed expression of Cas9 protein in Cas9/gNLRP3@M-N with a molecular weight of 180 kDa (Figure 2I). Neutrophil membrane markers such as integrin β2, macrophage-1 antigen (Mac-1), and lymphocyte function-associated antigen 1 (LFA-1) were present on all vesicle preparations, confirming that these vesicles possess the ability to migrate to inflammatory sites similar to neutrophils 24. Cas9 protein levels were significantly upregulated in Cas9/gNLRP3@M-N compared to other groups (Figure 2J).

The stability of Cas9/gNLRP3@M-N was assessed after 7 days of storage at 4 °C, and the hydrodynamic diameter and PDI remained unchanged (Figure 2K). Furthermore, Cas9 RNPs showed no signs of degradation (Figure S5A). Additionally, the encapsulation efficiency of the Cas9/gNLRP3@M-N complexes was approximately 40.77%, determined by fluorescence quantification. Given that the instability of Cas9 is a key limitation for clinical application, our membrane-based delivery method offers a promising solution. Overall, these results support the feasibility and effectiveness of Cas9/gNLRP3@M-N.

### Biosafety, biodistribution, and *in vivo* editing efficiency of Cas9/gNLRP3@M-N

**Figure 3:**
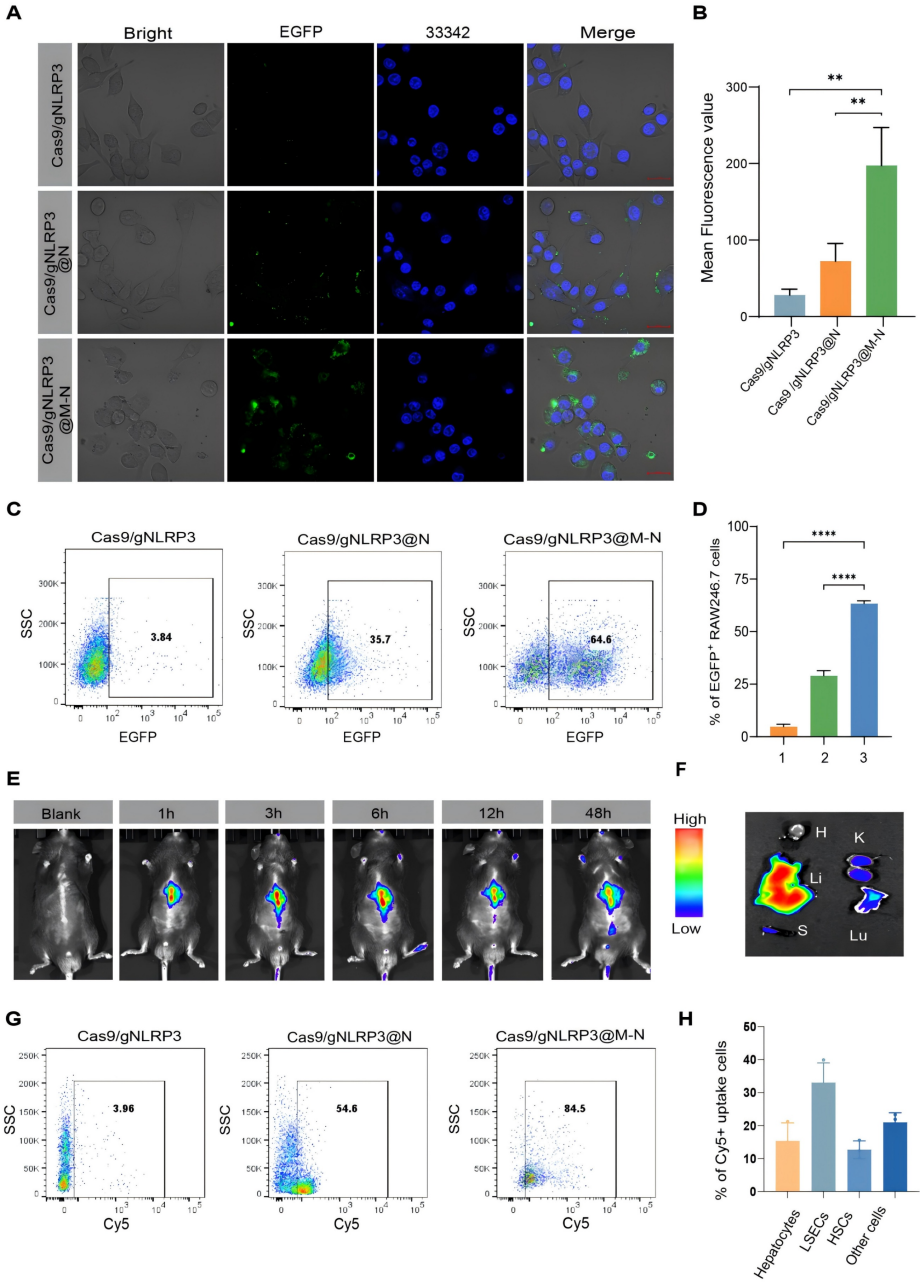
Biodistribution of Cas9/gNLRP3@M-N after internalization. A) CLSM analysis of Raw 264.7 cells after incubating with various EGFP-labeled nanoparticles. Cells grown with EGFP-labeled Cas9/gNLRP3 were used as controls. Scale bars: 20 μm. B) Fluorescence quantification. n = 3. C) Flow cytometry analysis of Raw 264.7 cells after incubation with various EGFP-labeled nanoparticles. D) Quantification of EGFP^+^ Raw246.7 cells across conditions. 1: Cas9/gNLRP3; 2: Cas9/gNLRP3@N; 3: Cas9/gNLRP3@M-N. n = 3. E, F) Real-time fluorescence distribution of *in vivo* Cy5-labeled Cas9/gNLRP3@M-N in the whole mice (top) and *in vitro* images of the liver and other major organs at 6 h after injection(bottom). H, heart; Lu, lung; Li, liver; K, kidney; S, spleen. G) Gating strategy for macrophages in liver tissue. Flow cytometry was used to evaluate macrophages (CD11b⁺ F4/80⁺) in mice treated with Cy5-labeled nanoparticles. H) Percentage of each hepatic cell type positive for Cy5-labeled Cas9/gNLRP3@M-N uptake. n = 3. Statistical significances were calculated via one-way ANOVA; **p < 0.01, ***p < 0.001, and ****p < 0.0001.

Before applying Cas9/gNLRP3@M-N for the treatment of inflammatory liver diseases, we assessed its cytotoxicity *in vitro*. RAW264.7 macrophage cell lines incubated with Cas9/gNLRP3@M-N showed no signs of cytotoxicity at concentrations used for *in vitro* experiments (Figure S6A), supporting the safety of further *in vitro* testing. Confocal laser scanning microscopy (CLSM) revealed efficient internalization of EGFP-labeled Cas9/gNLRP3@M-N by RAW264.7 cells, showing stronger fluorescence than cells treated with Cas9/gNLRP3 RNPs alone or Cas9/gNLRP3@N (Figure 3A, B). Flow cytometry analysis further confirmed significantly higher uptake of Cas9/gNLRP3@M-N compared to Cas9/gNLRP3 RNPs and Cas9/gNLRP3@N group (Figure 3C, D). These results suggest that membrane coating enhances the internalization of Cas9/gNLRP3 RNPs, and mannose modification further improves macrophage targeting.

A hemolysis assay demonstrated excellent hemocompatibility of Cas9/gNLRP3@M-N (Figure S6B). *In vivo* biosafety of Cas9/gNLRP3@M-N was evaluated in healthy C57BL/6 mice. The mice received high doses of Cas9/gNLRP3@M-N intravenously every other day for five treatments and were euthanized 7 days after the first injection. No deaths or significant weight changes were observed between the control and treated groups (Figure S7A). Blood chemistry analyses showed normal alanine aminotransferase (ALT) and aspartate aminotransferase (AST) levels (Figure S7B, C). Histological examination of organ sections stained with hematoxylin and eosin (H&E) showed no evidence of significant tissue damage (Figure S7D). Collectively, these results confirm the biocompatibility and safety of Cas9/gNLRP3@M-N *in vivo.*

We next assessed the *in vivo* distribution of Cas9/gNLRP3@M-N following systemic administration. LPS-induced inflammation mice were injected intravenously with Cy5-labeled Cas9/gNLRP3@M-N, and fluorescence distribution was tracked over time. The fluorescence intensity peaked at 6 hours post-injection, with the liver showing the highest fluorescence levels among major organs, likely due to its high macrophage population (Figure 3E, F). Flow cytometry analysis revealed that over 80% of Cy5-positive cells in the liver were CD11b^+^F4/80^+^ macrophages (Figure 3G), confirming macrophage-specific targeting of Cas9/gNLRP3@M-N. We further examined the biodistribution of Cas9/gNLRP3@M-N across various hepatic cell populations, including circulating monocyte-derived macrophages (Mo-Mφ) in the liver, Kupffer cells (KCs), parenchymal hepatocytes, liver sinusoidal endothelial cells (LSECs), Hepatic stellate cells (HSCs), and other non-parenchymal cells. The results confirmed that macrophages, particularly KCs, were the predominant cell type among the Cy5-positive cells (Figure S8 and 3H). We also evaluated Cas9/gNLRP3@M-N NLRP3 knockout efficiency *in vivo*. The results showed that the Cas9/gNLRP3@M-N group induced a robust indel frequency of 41.45% (Figure S9).

Collectively, these findings demonstrate that Cas9/gNLRP3@M-N achieves safe and efficient *in vivo* delivery, with high specificity for hepatic macrophages and robust genome-editing capability.

### Cas9/gNLRP3@M-N targeting NLRP3 ameliorates LPS/D-GalN-induced FH

**Figure 4:**
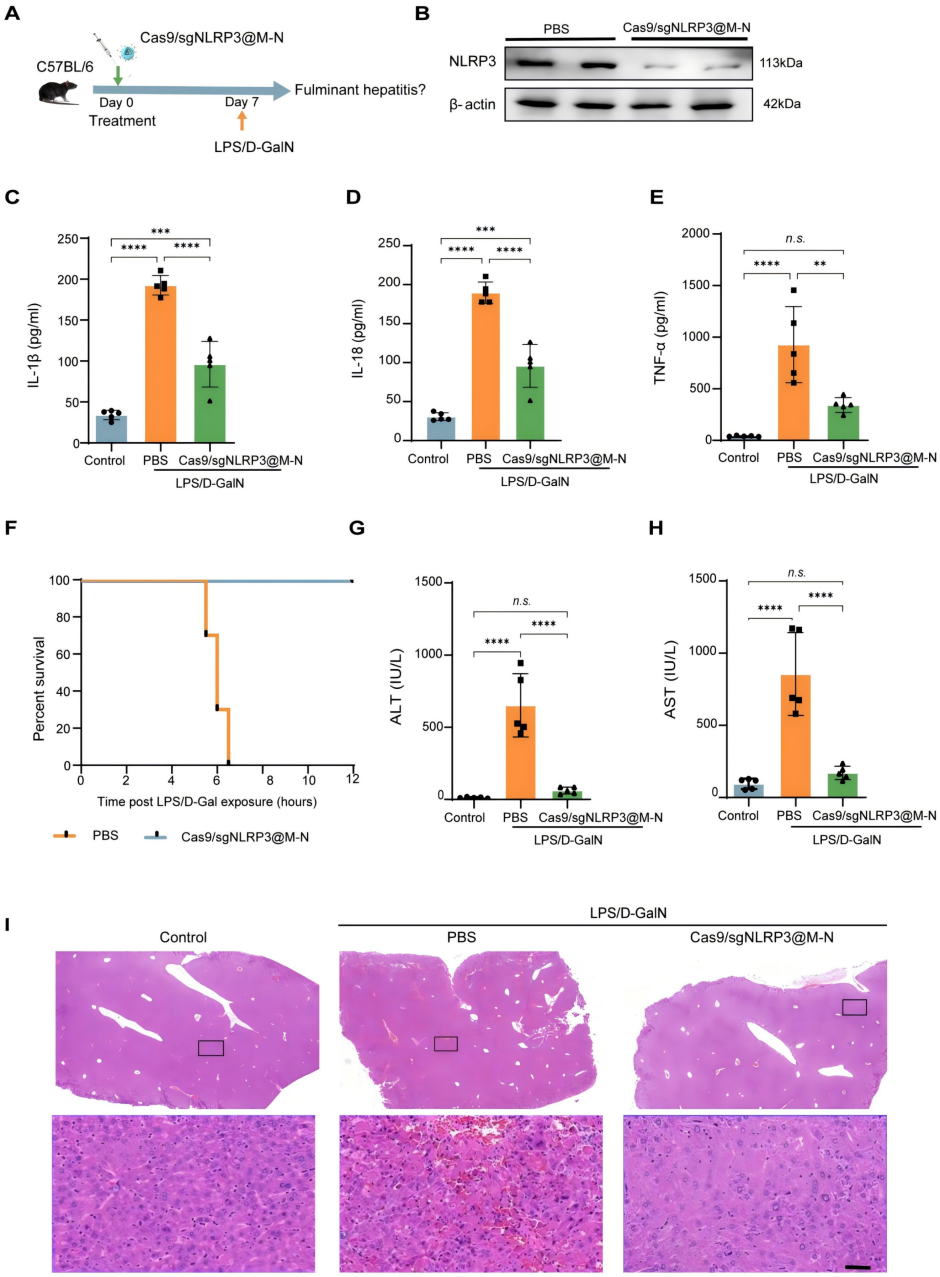
Cas9/gNLRP3@M-N suppresses LPS/D-GalN-induced acute inflammatory responses *in vivo*. A) Therapy scheme and B) immunoblot analysis of NLRP3 knockout efficiency in the liver. ELISA analysis. C) IL-1β, D) IL-18, E) TNF-α concentration in serum from mice 1.5 h after GalN/LPS treatment, pre-treated with Cas9/gNLRP3@M-N and then challenged with LPS/D-GalN. F) Mouse survival curve of LPS/D-GalN-induced fulminant hepatitis. n = 10 per group. The levels of G) AST and H) ALT in plasma were determined 6 h post LPS/D-Gal exposure, n = 5 per group. I) Liver tissue was collected 6 h after D-GalN/LPS treatment. Representative histological changes of the liver were obtained from mice of different groups. Significant histopathologic changes (such as inflammatory cell infiltration, congestion, necrosis, and degeneration) were observed in the D-GalN/LPS group; these pathologic alterations were ameliorated by the administration of Cas9/gNLRP3@M-N. Statistical significances were calculated via one-way ANOVA; **p < 0.01, ***p < 0.001, and ****p < 0.0001. n.s., no statistical significance.

We next evaluated the therapeutic efficacy of Cas9/gNLRP3@M-N in a model of acute inflammatory liver disease. Previous studies have shown that abnormal activation of the NLRP3 inflammasome plays a crucial role in the acute inflammatory response in fulminant hepatitis (FH), and that inhibiting its activation can alleviate FH-induced liver injury and inflammation 25. To test this, we pretreated mice with Cas9/gNLRP3@M-N or PBS before inducing FH with LPS/D-GalN (Figure 4A), a well-established model mimicking clinical features of acute liver failure.

As shown in Figure 4B, Cas9/gNLRP3@M-N treatment significantly reduced NLRP3 expression in the liver of FH model mice, indicating effective NLRP3 inflammasome suppression. We then assessed the downstream pro-inflammatory factors of the NLRP3 inflammasome in serum 1.5 hours after LPS/D-GalN injection, the time at which pro-inflammatory mediators typically peak. Compared to the PBS-treated group, Cas9/gNLRP3@M-N significantly reduced the levels of IL-1β, TNF-α, and IL-18 in the serum (Figure 4C-E), showing its protective effect against LPS/D-Gal-induced FH.

Remarkably, all FH model mice pretreated with Cas9/gNLRP3@M-N survived, while those pretreated with PBS died within 8 hours (Figure 4F). Additionally, Cas9/gNLRP3@M-N significantly reduced serum ALT and AST levels 6 hours after LPS/D-Gal injection, when liver injury peaked (Figure 4G, H). Histological analysis of liver tissue confirmed that Cas9/gNLRP3@M-N treatment greatly ameliorated LPS/D-Gal-induced extensive hemorrhagic necrosis, resembling healthy liver tissue (Figure 4I).

Two additional control groups—an empty neutrophil membrane vesicle group and a non-mannose-modified Cas9/gNLRP3@N group—failed to confer similar protective effects on liver inflammation, liver injury, or survival rates (Figure S10), highlighting the importance of mannose-mediated targeting.

In summary, a single injection of Cas9/gNLRP3@M-N significantly reduced LPS/D-Gal-induced inflammation, acute liver injury, and mortality in mice. These findings confirm that blocking the NLRP3 inflammasome can reverse LPS/D-GalN-induced FH. Encouraged by its efficacy in acute liver inflammation, we further explored its therapeutic potential in chronic liver inflammation.

### Cas9/gNLRP3@M-N targeting NLRP3 ameliorates GAN-induced hepatic steatosis and inflammation

**Figure 5:**
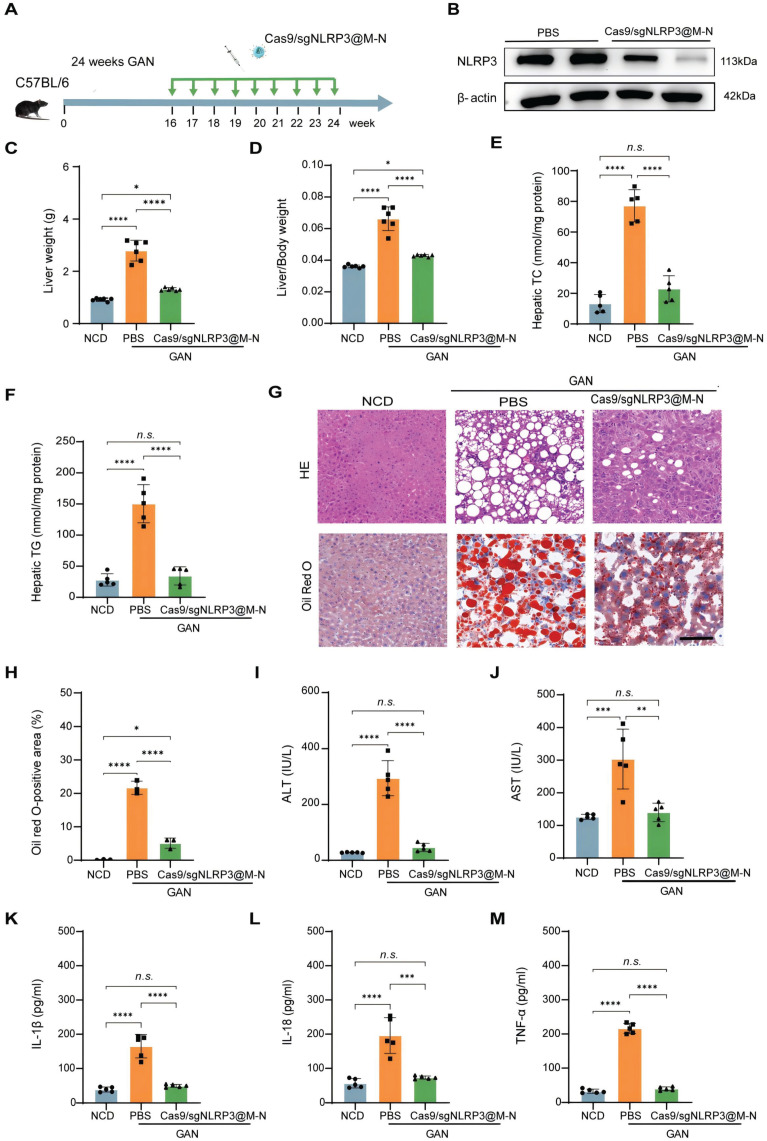
Cas9/gNLRP3@M-N suppresses hepatic steatosis and inflammation in GAN diet-fed mice. A) Schematic of therapeutic experiment design in mice. B) Immunoblot analysis of NLRP3 knockout efficiency in the liver. C) Liver weight and D) Liver/body weight ratio in the NCD, PBS, and Cas9/gNLRP3@M-N groups. n = 6 per group. E, F) Hepatic lipid (TG and TC) levels in the NCD, PBS, and Cas9/gNLRP3@M-N groups, n = 6 per group. G) H&E and Oil red O staining of liver tissues. Scale bar: 50 μm. H) The statistics of oil red O-positive areas. I) Serum ALT and J) AST in the NCD, PBS, and Cas9/gNLRP3@M-N group; n = 6 per group. ELISA analysis of the serum K) IL-1β, L) IL-18, M) TNF-α concentration in the NCD, PBS, and Cas9/gNLRP3@M-N groups. n = 6 per group. Statistical significances were calculated via one-way ANOVA; *p < 0.05, **p < 0.01, ***p < 0.001 and ****p < 0.0001. *n.s.,* no statistical significance.

To investigate whether Cas9/gNLRP3@M-N could also confer protection against chronic liver inflammation mediated by the NLRP3 inflammasome, we treated Gubra-Amylin NASH (GAN)-induced MASH mouse model with multiple intravenous injections of Cas9/gNLRP3@M-N following the treatment protocol shown in Figure 5A. Cas9/gNLRP3@M-N treatment significantly inhibited NLRP3 expression (Figure 5B).

Given that the NLRP3 inflammasome contributes to hepatocyte lipid metabolism disorders 27, we next examined whether inhibiting NLRP3 with Cas9/gNLRP3@M-N could attenuate hepatic steatosis in GAN diet-fed mice. After 24 weeks on the GAN diet, Cas9/gNLRP3@M-N-treated mice had a significantly lower liver weight than that of the PBS-treated control group and was comparable to the normal chow diet (NCD) group (Figure 5C). The liver-to-body weight ratio was also lower in the Cas9/gNLRP3@M-N-treated group compared to the PBS-treated control group (Figure 5D). Additionally, Cas9/gNLRP3@M-N significantly decreased hepatic triglyceride (TG) and total cholesterol (TC) levels in GAN diet-fed mice compared to the control group (Figure 5E, F). H&E and Oil Red O staining revealed that Cas9/gNLRP3@M-N treatment significantly reduced lipotoxicity-induced hepatocyte injury and hepatic lipid droplet accumulation (Figure 5G, H).

Furthermore, Cas9/gNLRP3@M-N significantly reduced the expression of genes involved in fatty acid synthesis and uptake (Srebp1, Fasn, Ppara, and Cpt1a) in GAN diet-fed mice (Figure S11). As lipotoxicity can cause hepatocyte damage and death, Cas9/gNLRP3@M-N-treated mice had lower serum ALT and AST levels than PBS-treated control mice (Figure 5I, J).

We then assessed the effect of Cas9/gNLRP3@M-N on MASH-associated inflammation. Immunohistochemistry showed a marked reduction in hepatic infiltration of inflammatory cells, such as neutrophils (MPO) and macrophages (F4/80), in Cas9/gNLRP3@M-N-treated mice compared to PBS-treated control mice (Figure S12). Quantitative real-time PCR confirmed that Cas9/gNLRP3@M-N treatment suppressed the expression of inflammatory genes (IL-1β, IL-18, TNF-α, and IL-6) (Figure S13). Additionally, serum levels of inflammatory cytokines (IL-1β, IL-18, and TNF-α) were significantly reduced in GAN diet-fed mice treated with Cas9/gNLRP3@M-N (Figure 5K-M).

In conclusion, these results demonstrate that Cas9/gNLRP3@M-N mitigates GAN diet-induced hepatic lipid dysfunction and inflammation by inhibiting NLRP3 inflammasome activation in macrophages, which in turn reduces the accumulation of inflammatory cytokine-induced fat in the liver.

### Cas9/gNLRP3@M-N targeting NLRP3 ameliorates CDAHFD-induced hepatic inflammation and fibrosis

**Figure 6:**
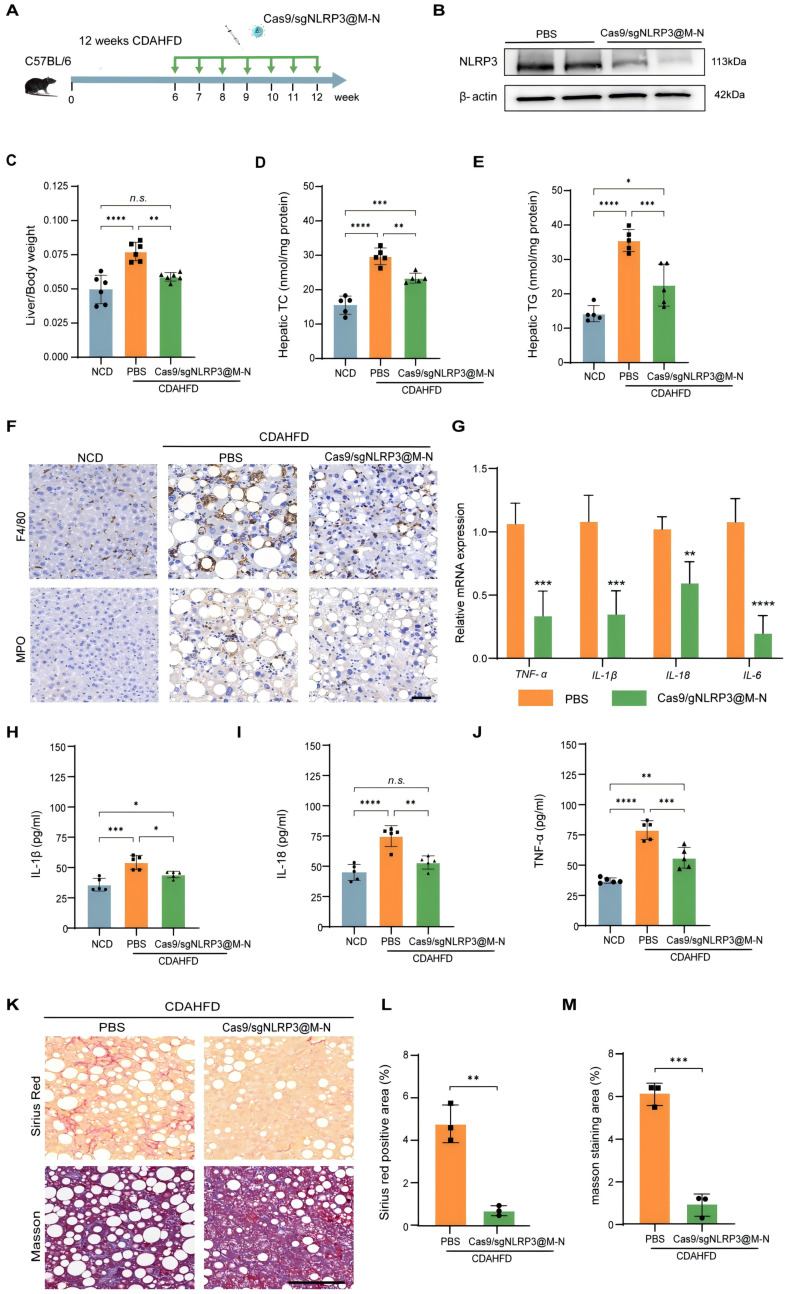
Cas9/gNLRP3@M-N attenuated hepatic inflammation and fibrosis in CDAHFD diet-fed mice. A) Schematic of the experimental procedure. B) Immunoblot analysis of NLRP3 knockout efficiency in the liver. C) Liver/body weight ratio of mice. n = 6 per group. D, E) Hepatic lipid (TC and TG) levels. n = 6 per group. F) Representative histological results of liver sections stained with F4/80, MPO. Scale bar: 50 μm. G) Quantitative real-time PCR analysis of the transcript levels of genes related to inflammation. Gene expression was normalized to GAPDH mRNA levels. n =5 per group. ELISA analysis of the H) IL-1β, I) IL-18, J) TNF-α concentration in serum. K) Representative images of Sirius red staining and Masson staining. Scale bar: 100μm. Percentage of L) Sirius Red, and M) Masson staining positive area in randomly selected fields from each specimen by computerized image analysis. *p < 0.05, **p < 0.01, ***p < 0.001 and ****p < 0.0001. (C-E and H-I, one-way ANOVA; G, L, and M, two-tailed t-test). *n.s.,* no statistical significance.

To further evaluate the therapeutic potential of Cas9/gNLRP3@M-N in chronic liver injury, we used a more severe MASH model induced by a choline-deficient, L-amino acid-defined, high-fat diet (CDAHFD) (Figure 6A). This model mimics advanced human MASH with pronounced inflammation and fibrosis 26.

Pretreatment with Cas9/gNLRP3@M-N significantly inhibited NLRP3 activation (Figure 6B). Consistent with the findings from the GAN-induced MASH model, Cas9/gNLRP3@M-N treatment significantly reduced CDAHFD-induced increases in liver weight and the liver-to-body weight ratio (Figure S14, 6C). Histological analysis, including H&E and Oil Red O staining, revealed that Cas9/gNLRP3@M-N treatment reduced microvesicular and macrovesicular fat deposition as well as hepatocyte ballooning (Figure S15). Additionally, Cas9/gNLRP3@M-N significantly attenuated CDAHFD-induced expression of lipid-related genes (Srebp1, Fasn, Ppara) (Figure S16) and reduced hepatic TG, TC, serum ALT, and AST levels (Figure 6D, E, S17). Taken together with the GAN model data, these results indicate that Cas9/gNLRP3@M-N can mitigate hepatocyte injury, hepatic steatosis, and dysregulation of fatty acid metabolism under metabolic stress conditions.

We next assessed the effects of Cas9/gNLRP3@M-N on MASH-related inflammation and fibrosis. Cas9/gNLRP3@M-N treatment significantly reduced CDAHFD-induced inflammatory cell infiltration in the liver (Figure 6F). Serum pro-inflammatory cytokine levels and hepatic pro-inflammatory cytokine gene expression were notably downregulated by Cas9/gNLRP3@M-N treatment (Figure 6G-J). Sirius Red, Masson, and α-SMA staining demonstrated that Cas9/gNLRP3@M-N treatment markedly alleviated CDAHFD-induced hepatic fibrosis (Figure 6K-M, S18). These findings suggest that Cas9/gNLRP3@M-N effectively inhibits liver inflammation and fibrosis in a 12-week CDAHFD-induced NASH model.

Together, these results demonstrate that Cas9/gNLRP3@M-N can alleviate inflammation and fibrosis in advanced MASH. Its strong anti-inflammatory effects and ability to modulate fibrotic progression suggest that NLRP3 inflammasome inhibition may be a promising therapeutic strategy not only for prevention but also for reversal of liver fibrosis.

## Discussion

In this study, we developed a novel macrophage-targeted genome editing strategy for delivering CRISPR/Cas9 RNPs using mannosylated neutrophil vesicles. Our therapeutic strategy focuses on the NLRP3 inflammasome, a critical mediator of both acute and chronic liver inflammation, primarily acting through macrophages. The NLRP3 gene plays a pivotal role in liver macrophage-driven inflammation 28. However, most existing NLRP3 inhibitors tend to be non-specific and often demonstrate limited efficacy 29. In contrast, the CRISPR/Cas9 system offers a more precise and potent method to target and eliminate NLRP3-dependent inflammation directly.

Our research demonstrated that delivering CRISPR/Cas9 RNPs targeting NLRP3 via mannosylated neutrophil vesicles enables efficient macrophage-specific targeting and significantly alleviates NLRP3-dependent inflammation. Direct delivery of CRISPR/Cas9 RNPs avoids transgene integration and minimizes off-target effects, providing a distinct advantage compared to other formulations 30. However, due to their negative charge and large size, RNPs face substantial barriers in crossing cell membranes and often exceed the payload capacity of viral vectors. These challenges underscore the need for alternative, non-viral delivery strategies, including physical and chemical approaches, to achieve effective therapeutic genome editing 31.

In recent years, delivery systems based on extracellular vesicles (EVs) have emerged as promising delivery vehicles that bridge the benefits of viral and nonviral delivery methods 32. Membrane vesicles have properties similar to those of natural EVs, allowing a scalable alternative with higher yields and customizable features. Our delivery system leverages neutrophil membrane vesicles to replicate immune cell functions, incorporating immunomodulatory molecules to target the site of inflammation precisely. In addition, we functionalized the vesicle surface with targeting ligands by inserting DSPE-conjugated mannose (Man) and polyethylene glycol (PEG), enhancing macrophage-specific delivery through mannose receptor-mediated uptake. This strategy is based on the lipid structure and fluidity of the cell membrane and has the advantage of being convenient and easy to implement 33.

We demonstrated both *in vitro* and *in vivo* that Cas9/gNLRP3@M-N was efficiently internalized by macrophages. In the *in vivo* studies, macrophages, due to their robust phagocytic activity, could effectively internalize the construct, confirming cell-type specificity. Our approach enhances delivery efficiency while minimizing cytotoxicity and immunogenicity. Since NLRP3 functions primarily in macrophages, disrupting the NLRP3 gene in other cell types does not result in predictable side effects. Moreover, macrophages typically have a lifespan of 6 to 16 days, meaning NLRP3 disruption in these cells is reversible as macrophages are actively replaced and do not lead to permanent inhibition of NLRP3 12. Therefore, Cas9/gNLRP3@M-N holds great potential for the clinical treatment of NLRP3-dependent inflammatory diseases. Cas9/gNLRP3@M-N effectively attenuated fulminant hepatitis induced by LPS/D-GalN by dampening the associated acute inflammatory response and liver injury. Furthermore, Cas9/gNLRP3@M-N successfully alleviated chronic inflammation, hepatic steatosis, and fibrosis in MASH in two different animal models. Taken together, these results highlight Cas9/gNLRP3@M-N as a promising therapeutic platform for macrophage-targeted gene editing and support its potential application in the treatment of NLRP3-mediated inflammatory diseases.

## Conclusion

In summary, we developed a novel macrophage-targeting genome-editing strategy by delivering CRISPR/Cas9 RNPs via mannosylated neutrophil vesicles. The resulting construct, Cas9/gNLRP3@M-N, specifically targets the NLRP3 gene, which is key in liver macrophage inflammation. Treatment with Cas9/gNLRP3@M-N significantly reduced acute inflammatory responses and liver injury in the FH model, and alleviated chronic inflammation, hepatic steatosis, and fibrosis in MASH in two distinct animal models. This work demonstrates a proof of concept for macrophage-specific gene therapy in liver inflammation-related diseases. As we move toward clinical translation of Cas9/gNLRP3@M-N, considerations for large-scale production and delivery must be addressed. Established protocols for clinical-grade neutrophil production provide a reliable source of membrane material, while ongoing advancements in gene therapy manufacturing and regulatory standards offer a viable path toward therapeutic development.

## Experimental Section/Methods

### Neutrophil membrane preparation

Neutrophils were isolated from mouse bone marrow using a neutrophil isolation kit (MACS). Neutrophil purity was confirmed through flow cytometry (Beckman, Cytoflex S). Neutrophil membranes were then prepared as previously described 34. Subsequently, collected neutrophils were washed three times with phosphate-buffered saline (PBS) by centrifugation at 500g. The cells underwent three freeze-thaw cycles followed by centrifugation at 700 g for 10 minutes at 4 °C. The cell suspensions were then centrifuged at 14,000 g for 30 minutes at 4 °C, and the resulting pellet was stored at -80 °C for further use.

### Purification of spCas9 protein

The plasmid pET-NLS-Cas9-6×His (Addgene ID: 62934) was transformed into *Escherichia coli* BL21 Star (DE3). The transformed bacteria were pre-cultured in 10 mL of LB (Amp) medium for 16 hours at 37 °C. The pre-culture was transferred to 1L of LB medium and incubated until the OD600 reached 0.6 at 37 °C. Protein expression was induced with 0.5 mM isopropyl β-D-1-thiogalactopyranoside (IPTG, Sigma, USA) at 20 °C for 16 hours. The cells were harvested and resuspended in 25 mL of lysis buffer containing 10 mM protease inhibitor (PMSF, Sigma, USA). The suspension was sonicated for 20 minutes, and cellular debris was removed by centrifugation at 12,000 rpm for 30 minutes at 4 °C. spCas9 protein was purified using HisSep Ni-NTA MagBeads (Yeasen, China) following the manufacturer's protocol. The purity of spCas9 was assessed using SDS-PAGE, and protein concentrations were measured using a BCA assay kit.

### gRNA design and synthesis

spCas9 gRNAs were synthesized using an *in vitro* transcription kit by T7 RNA polymerase, following the manufacturer's instructions. The transcription template, containing the T7 promoter, target, and gRNA sequence, was generated through polymerase chain reaction (PCR). The sequences are provided in Table S2.

### Synthesis of DSPE-PEG-Man

DSPE-PEG-NH2 was dissolved in 10 mL of 0.1 M PBS (pH 9.0) and added dropwise to mannose (10 mg in 1 mL of DMSO). The mixture was stirred at room temperature for 24 hours. After overnight stirring, the solution was dialyzed (MWCO: 3 kDa) against deionized water for 2 days and then freeze-dried to yield DSPE-PEG-Man. The structure was confirmed using ^1^H NMR.

### Cas9/gNLRP3@M-N synthesis

For membrane coating, 50 µg of neutrophil membranes were mixed with Cas9 RNPs at a 1:1 RNP complex-to-membrane protein weight ratio. The mixture was sonicated for 5 minutes in a bath sonicator and then passed through an extruder (Avanti Polar Lipids, Inc.) with polycarbonate membranes of 800 nm, 400 nm, and 200 nm pore sizes. The final product was incubated with 50 µg of DSPE-PEG-Man for 30 minutes. Then, the mixture was centrifuged at 14,000 g for 30 minutes at 4^o^C to remove free Cas9 RNPs and DSPE-PEG-Man.

### Cas9/gNLRP3@M-N characterization

The hydrodynamic size and surface charge of Cas9/gNLRP3@M-N were measured using dynamic light scattering (Zetasizer Nano ZS, Malvern Instruments Ltd., UK). The morphology and membrane integrity of Cas9/gNLRP3@M-N were assessed via transmission electron microscopy (TEM) (HT7700, Hitachi, Japan) after staining with a 5% phosphotungstic acid negative staining solution. Neutrophil-specific surface markers were identified on neutrophil membranes and Cas9/gNLRP3@M-N by western blotting, and membrane protein transfer was confirmed by sodium dodecyl sulfate-polyacrylamide gel electrophoresis (SDS-PAGE). The encapsulation efficiency (EE%) of Cas9 was determined using fluorescence quantification. Cas9 was labeled with EGFP, and a standard curve was established using a fluorescence spectrophotometer (Figure S5B). The encapsulation efficiency was calculated using the formula EE (%) =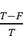
×100%, where T is the total protein content and F is the free protein content.

### Cell culture

RAW264.7 cells were obtained from the China Center for Typical Culture Collection in Wuhan, China. The cells were cultured in DMEM (Gibco) supplemented with 10% fetal bovine serum (Wisent) and 1% penicillin-streptomycin (Gibco) and incubated at 37 °C in a 5% CO_2_ atmosphere. Before incubation with nanoassemblies, the cells were washed twice with PBS.

### *In vitro* cellular uptake

RAW 264.7 cells (1×10^5^) were seeded in confocal dishes (glass-bottom culture dishes, MatTek) and incubated for 24 hours. EGFP-labeled Cas9 protein was used to track the uptake of Cas9/gNLRP3 and other nanoparticles. The cells were incubated with various EGFP-labeled nanoparticles (Cas9 concentration: 20 μg/mL) for 6 hours. After washing with PBS (twice), the cell nuclei were stained with Hoechst 33342 for 15 minutes at 37 °C, followed by another PBS wash. Cells were then fixed with 4% paraformaldehyde (PFA) and visualized using a confocal laser scanning microscope (Carl Zeiss LSM880).

### Hemolysis assay

The hemocompatibility of Cas9/gNLRP3@M-N was evaluated using a hemolysis assay. Fresh mouse whole blood was collected with heparin, centrifuged at 3000 rpm for 10 minutes to separate the plasma, and the red blood cells (RBCs) were washed with PBS before being resuspended in a 5% RBC suspension. Cas9/gNLRP3@M-N and the neutrophil vesicle were incubated with this suspension at 37 °C for 2 hours. Distilled water was used as a positive control, and PBS was used as a negative control. Following incubation, samples were centrifuged, and the supernatants were analyzed for hemolysis by comparing their colors.

### *In vivo* fluorescence imaging and biodistribution of nanoparticles

C57BL/6 mice were randomly assigned to two groups (n = 3). One group served as the control for data normalization, and the other group received an intravenous injection of 200 µL of PBS containing 5 mg/mL of Cy5-labeled nanoparticles. Fluorescence images were captured at different time points (1 h, 3 h, 6 h, 12 h, and 48 h) using the IVIS Spectrum system (PerkinElmer). At 6 hours post-injection, mice were euthanized, and major organs (heart, liver, spleen, lung, and kidney) were collected for *ex vivo* fluorescence imaging.

### Biodistribution of Cas9/gNLRP3@M-N within different hepatic cell types

Liver cell suspensions, including multiple hepatic cell types, were obtained using a previously described method involving *in situ* perfusion, collagenase digestion, filtration, and centrifugation 35. The suspension was centrifuged at 50 g for 3 min at 4 °C to collect hepatocytes, and the supernatant containing nonparenchymal cells (NPCs) was collected. For further separation, NPCs were layered onto a two-step Percoll gradient and centrifuged at 1400 g for 11 min at 4 °C without brake. The middle layer containing NPCs was collected and washed. Flow cytometry analysis assessed Cas9/gNLRP3@M-N uptake in different hepatic cell populations, including hepatocytes, KCs, LSECs, and Mo-Mφ.

### T7E1 assays and Sanger sequencing

To evaluate genome editing efficiency at the target loci, genomic DNA was extracted from the collected cells using the FastPure Cell/Tissue DNA Isolation Mini Kit (Vazyme Biotech). T7 Endonuclease I (T7E1) assay and Sanger sequencing were then performed to detect insertions and deletions (indels) at the target sites, following previously described protocols 36.

### Animal models

C57BL/6J mice were housed under pathogen-free conditions in temperature-controlled rooms (25 ± 5 °C) with a 12-hour light/dark cycle and ad libitum access to food and water. To induce fulminant hepatitis, mice were injected intraperitoneally with LPS (10 μg/kg) and D-GalN (700 mg/kg). Seven days before the injections, mice were pretreated with 100 μg of Cas9/gNLRP3@M-N.

For the MASH model, 6-8week-old male C57BL/6 mice were continuously fed either a CDAHFD diet (TP3622648, Trophic Diets, Nantong, China) or a GAN diet (TP2834044, Trophic Diets, Nantong, China) for 12 or 24 weeks. Mice on a normal chow diet (NCD) (D12450J, Research Diets, New Brunswick, NJ) served as controls. Cas9/gNLRP3@M-N complexes were administered weekly throughout the experiment at a dose of 100 μg of Cas9 protein. All animal procedures were approved by the Ethics Committee of Anhui Medical University.

### Biochemical assays

ALT and AST levels were measured using a Mindray automatic biochemistry analyzer, following the manufacturer's protocols (Shenzhen Mindray Biomedical Electronics Co., Ltd.). Hepatic TG and TC levels were quantified using the TG Enzymatic Determination Kit and TC Enzymatic Determination Kit.

### Quantitative PCR analysis

Total RNA was extracted using TRIzol reagent (Invitrogen, USA), and mRNA was reverse-transcribed into cDNA using the PrimeScript™ RT reagent kit (Takara, Japan), following the manufacturer's protocol. Quantitative PCR (qPCR) was conducted with SYBR Green (Roche, Indianapolis, IN, USA) to quantify mRNA expression levels. GAPDH was used as the housekeeping gene for normalization, and the data were presented as fold changes relative to basal levels. The forward and reverse primer sequences for each gene are listed in Table S1.

### ELISA assay

Serum levels of mouse IL-1β, IL-18, and TNF-α were measured using ELISA kits, following the manufacturer's instructions. The ELISA detection kits for IL-1β, IL-18, and TNF-α were purchased from Wuhan Boster Biological Technology, Ltd.

### Histopathological analysis

For histological examination, liver tissues were fixed in 4% paraformaldehyde (PFA) and embedded in paraffin for hematoxylin and eosin (H&E), Masson, and Sirius Red staining. Oil Red O staining was performed on frozen liver sections embedded in optimal cutting temperature (OCT) compound. Immunohistochemical staining for F4/80, MPO, and α-SMA was performed on paraffin-embedded liver tissues fixed in 4% PFA. All slide images were scanned with an automatic digital slide scanner (Pannoramic MIDI, 3DHISTECH, Hungary) and analyzed using CaseViewer software.

### Statistical analysis

All statistical analyses were performed using GraphPad Prism software (version 6.0). Biological replicates were utilized for all experiments. Data were analyzed using either Student's t-test (two-tailed) or one-way analysis of variance (ANOVA). Results are expressed as mean ± SD. Statistical significance was defined as a P-value < 0.05. Significance levels are indicated as follows: N.S. (not significant), *P < 0.05, **P < 0.01, ***P < 0.001, and ****P < 0.0001.

## Supplementary Material

Supplementary figures and tables.
