# Supplementary material for: Mannosylated neutrophil vesicles targeting macrophages alleviate liver inflammation by delivering CRISPR/Cas9 RNPs
Source: Theranostics. 2025 May 8;15(13):6221–35. doi: 10.7150/thno.107791 (PMC12159820; doi:10.7150/thno.107791)
Supplement: Supplementary file 1 — Supplementary figures and tables. [file thnov15p6221s1.pdf]

Supporting Information

Mannosylated neutrophil vesicles targeting macrophages alleviate liver inflammation by delivering CRISPR/Cas9 RNPs

Supplementary figures

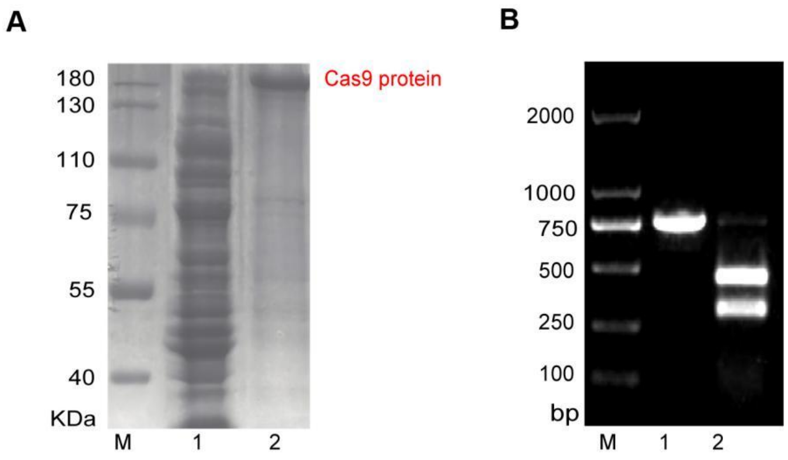

Figure S1. A) Purification and detection of Cas9 protein, M:Marker, 1:Bacterial lysate, 2:After purification. B) The cleavage activity of Cas9 protein was determined by agarose gel electrophoresis, M:Marker, 1: DNA substrate in the kit, 2: DNA substrate and gRNA in the kit + purified Cas9 protein.

\

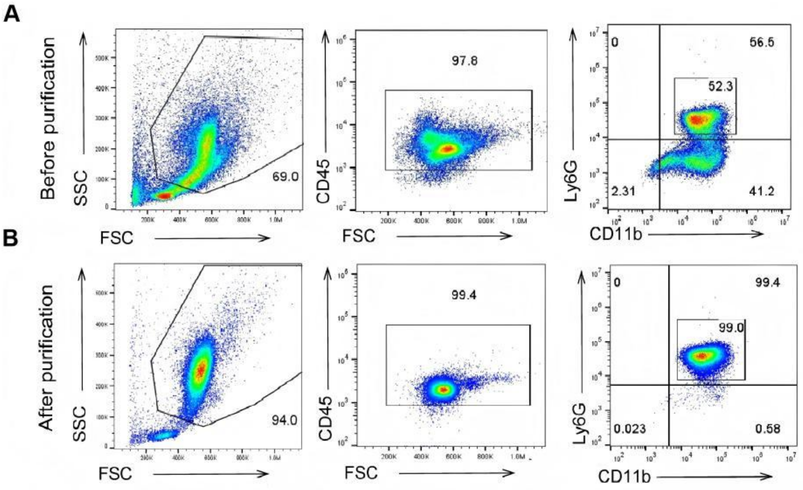

Figure S2. Flow cytometry was used to detect mouse bone marrow neutrophils(CD45$^+$CD11b$^+$ Ly6G$^+$). A) Before purification, B) After purification.

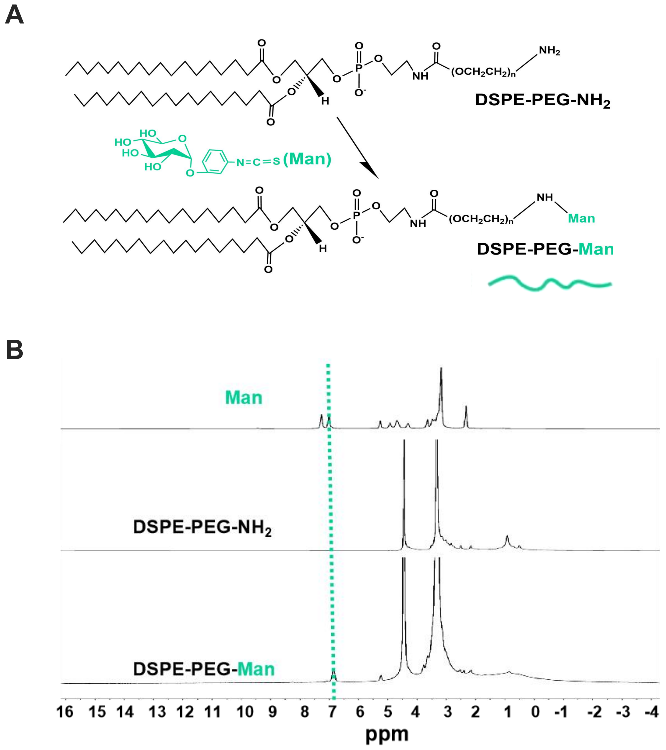

Figure S3. A)Synthesis route and B) nuclear magnetic resonance (H^1 NMR) detection of DSPE-PEG-Man .

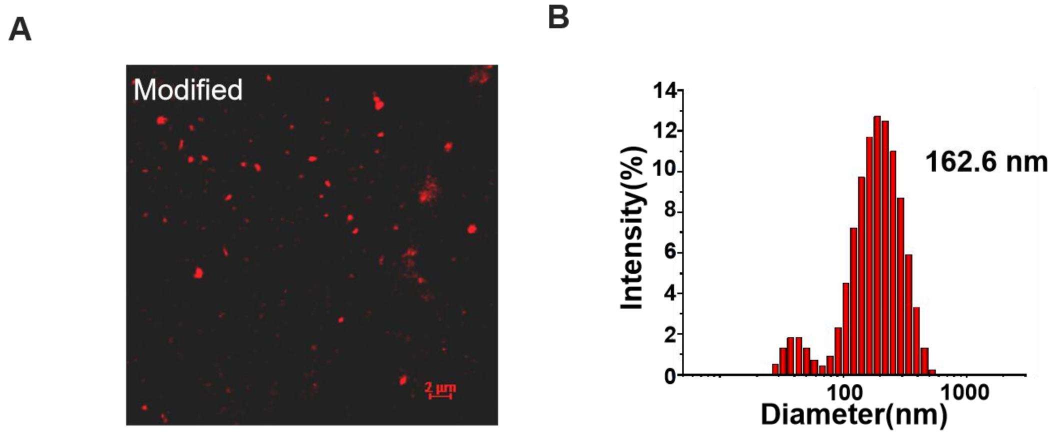

Figure S4. A) Confocal microscopy observation of neutrophil vesicle modified with DSPE-PEG-Cy5. scale bar: 2 μm. B) The hydrodynamic diameter of empty neutrophil vesicle were analyzed by dynamic light scattering (DLS).

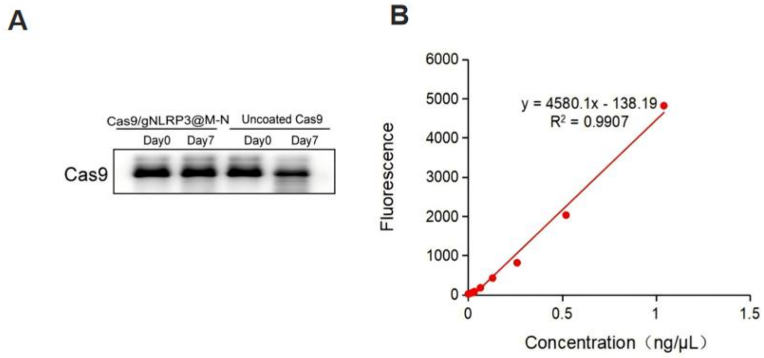

Figure S5. A) WB analysis of Cas9/gNLRP3@M-N and Cas9 before and after storage at 4℃ for 7 days. B) Standard concentration curve.

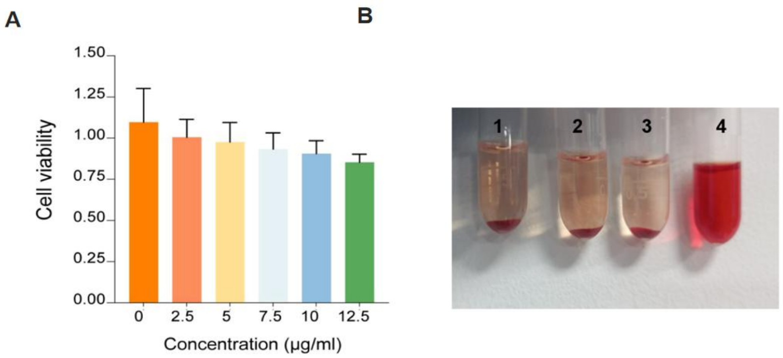

Figure S6. A) The cytotoxicity of different concentrations of Cas9/gNLRP3@M-N to RAW264.7 cells was detected by MTT assay. No statistical significance between groups. B) Images and hemolysis ratios of erythrocyte suspensions after treatment. 1: PBS; 2: Neutrophil vesicle; 3: Cas9/gNLRP3@M-N; 4: Water.

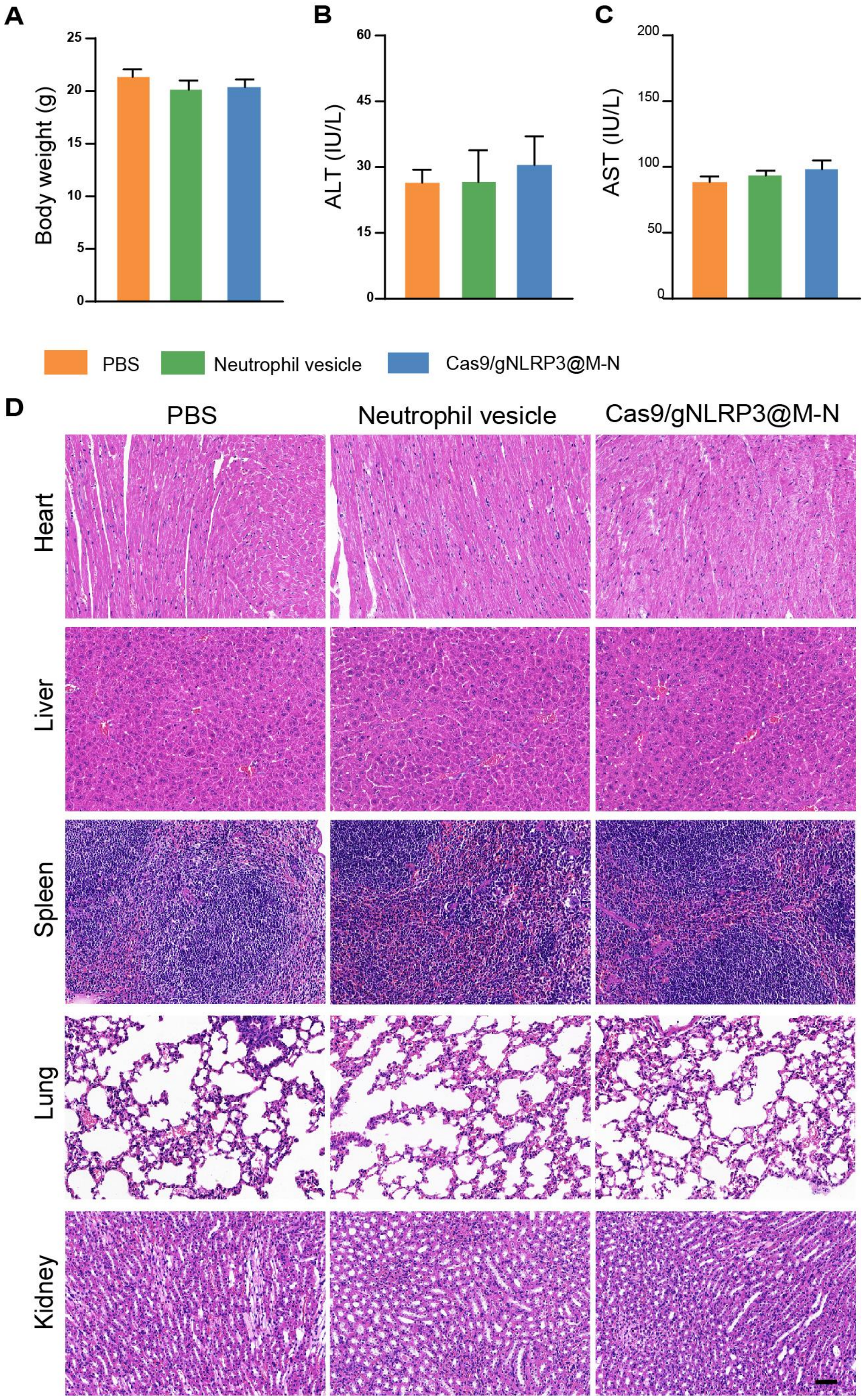

Figure S7. A) Body weight, and serum levels of B) ALT, C) AST in healthy mice treated with PBS, neutrophil vesicle, and Cas9/gNLRP3@M-N groups. No statistical significance between groups. d)H&E staining of histological sections from major organs, including the heart, liver, spleen, lungs, and kidneys. scale bar: 50 μm; n = 5 mice per group.

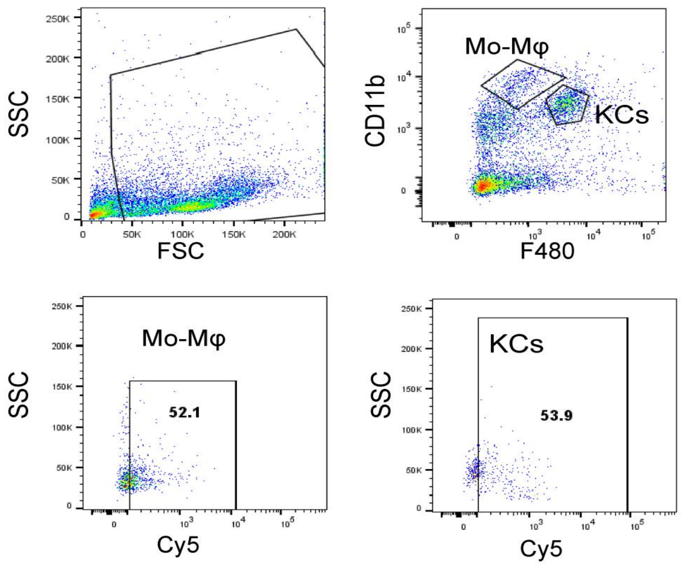

Figure S8. Flow cytometric identification of Kupffer cells (KCs) and monocyte-derived macrophages (Mo-Mφ) in mouse liver.

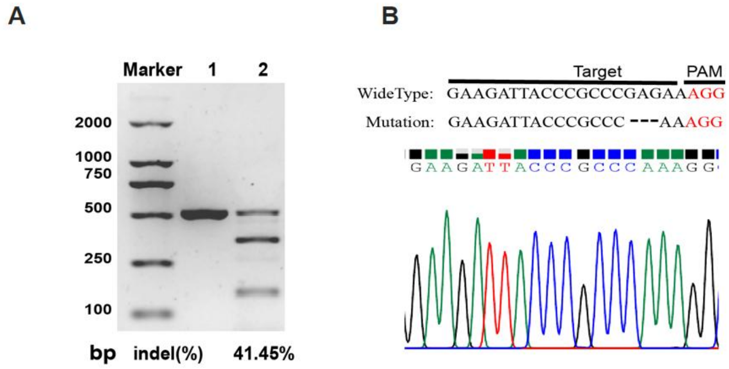

Figure S9. *In vivo* evaluation of NLRP3 gene editing efficiency. A) T7E1 assay of liver macrophages from mice treated with different formulations. Lane 1: substrate DNA. Lane 2: Cas9/gNLRP3@M-N. B) Sanger sequencing showing wild-type and mutant sequences with a deletion at the target site. PAM in red.

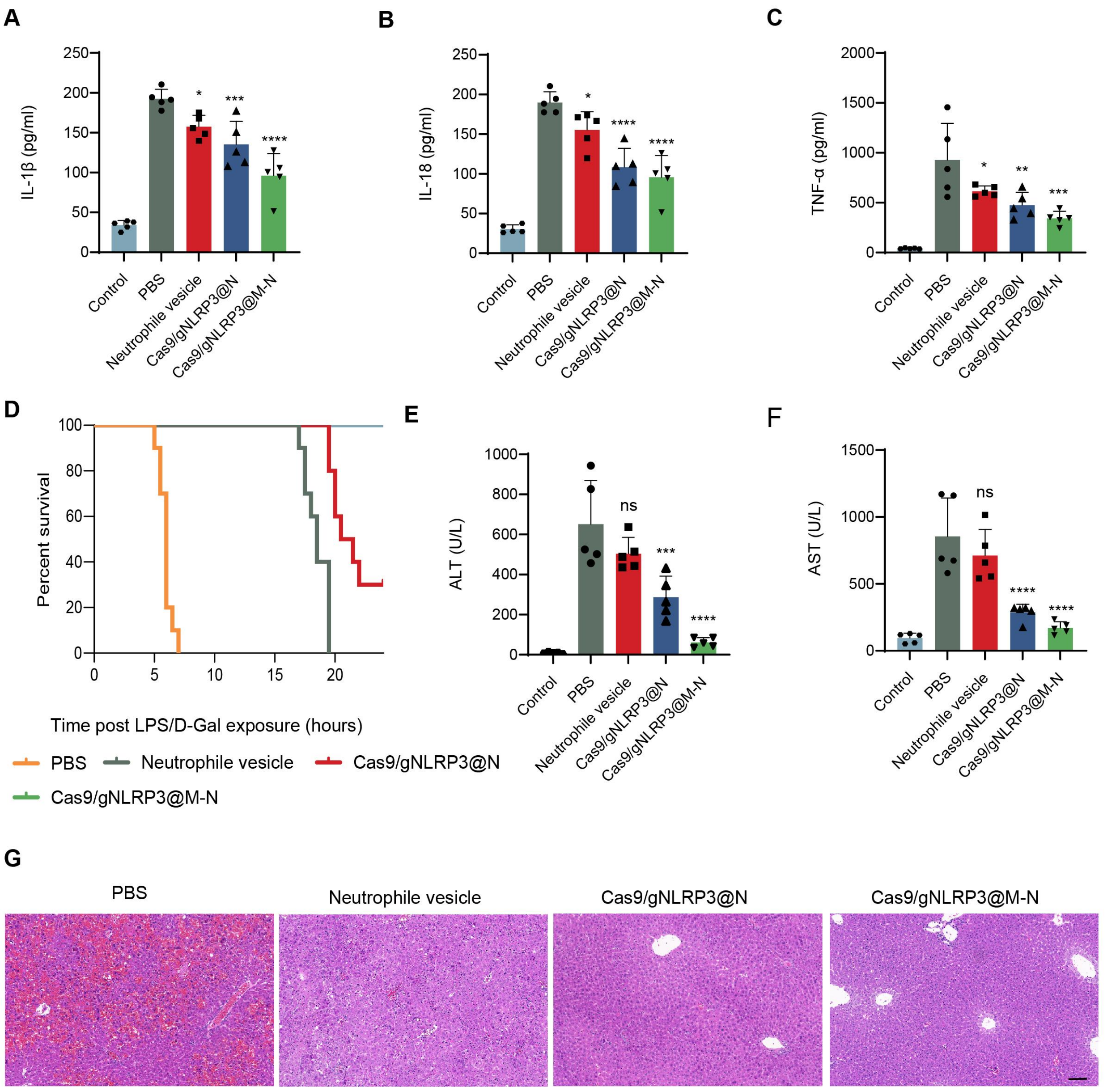

Figure S10. A) IL-1β, B) IL-18, C) TNF-α concentration in serum from mice 1.5 h after GalN/LPS treatment, pre-treated with different groups and then challenged with LPS/D-GalN. n=5 per group. D) Mouse survival curve of LPS/D-GalN-induced fulminant hepatitis. n=10 per group. The levels of E) AST and F) ALT in plasma were determined 6 h post LPS/D-Gal exposure. n=5 per group. G) Liver tissue was collected 6 h after D-GalN/LPS treatment. Representative histological changes of the liver were obtained from mice of different groups. Significant histopathologic changes (such as inflammatory cell infiltration, congestion, necrosis, and degeneration) were observed in the D-GalN/LPS group; Statistical significances were calculated via the one-way ANOVA; $**p < 0.01$, $***p < 0.001$ and $****p < 0.0001$. n.s., no statistical significance.

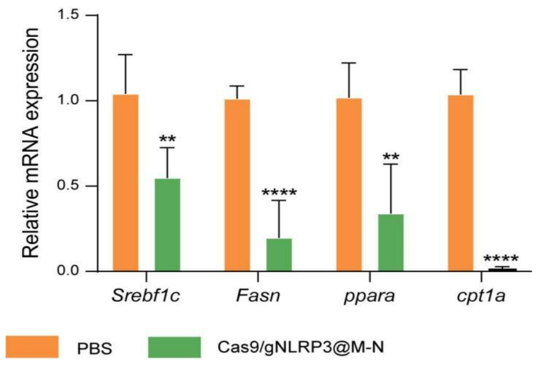

Figure S11. Relative mRNA levels of genes associated with lipid metabolism (*Srebp1, Fasn, Ppara* and *cpt1a*). n = 5 mice per group. The mRNA expression levels of the genes were normalized to GAPDH. Statistical significances were calculated via the Student's t-test; $**p < 0.01$, $****p < 0.0001$.

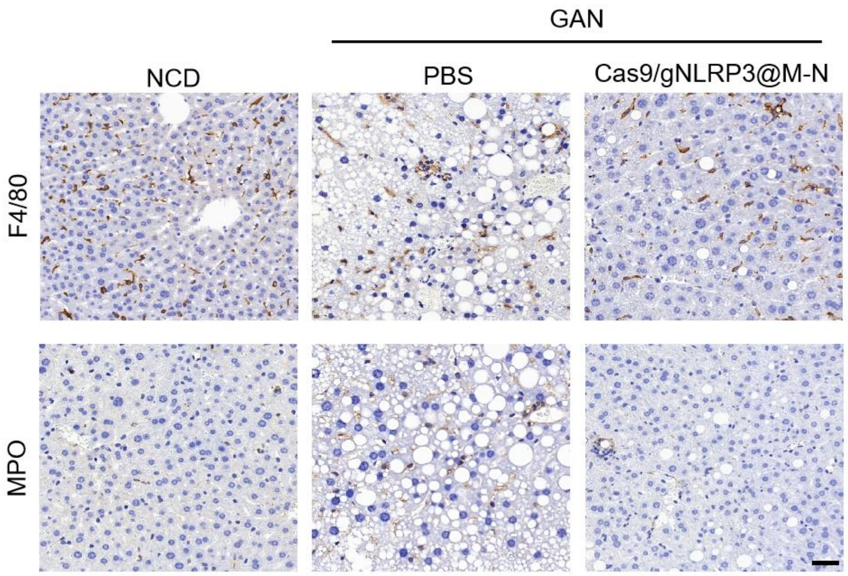

Figure S12. Representative histological results of liver sections stained with F4/80, MPO, scale bar: 50 μm.

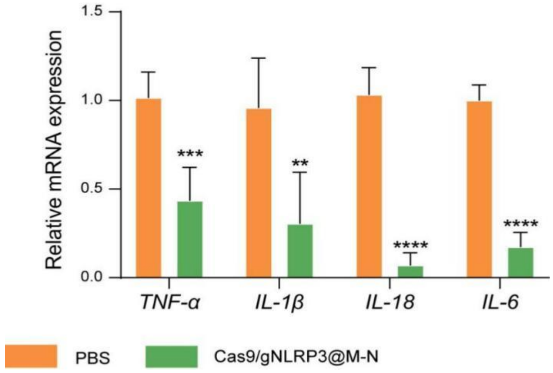

Figure S13. Relative mRNA levels of genes associated with inflammatory genes (*IL-1β, IL-18* ,*TNF-α* and *IL-6*). n = 5 mice per group. The mRNA expression levels of the genes were normalized to GAPDH. Statistical significances were calculated via the Student's t-test; $^{**}p < 0.01$, $^{***}p < 0.001$ and $^{****}p < 0.0001$.

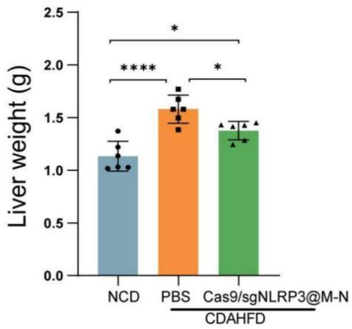

Figure S14. Liver weight of mice in the NCD, PBS, and Cas9/gNLRP3@M-N groups. n = 6 per group. Statistical significances were calculated via the one-way ANOVA ; $*p < 0.05$, $****p < 0.0001$.

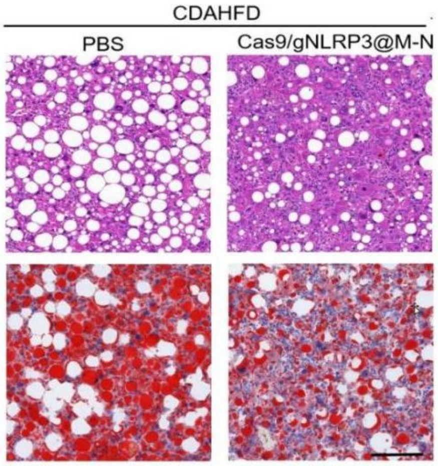

Figure S15. Representative H&E staining, Oil red O staining in the liver sections. scale bar: 100 µm, n = 6 per group.

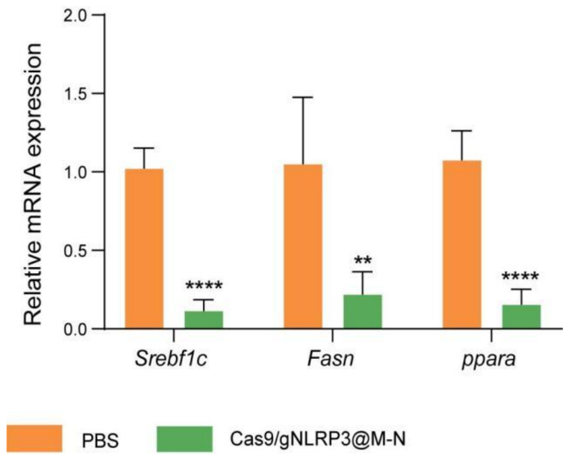

Figure S16. Relative mRNA levels of genes associated with lipid metabolism (*Srebp1, Fasn, Ppara*). n = 6 mice per group. The mRNA expression levels of the genes were normalized to GAPDH. Statistical significances were calculated via the Student's t-test; $^{**}p < 0.01$, $^{****}p < 0.0001$.

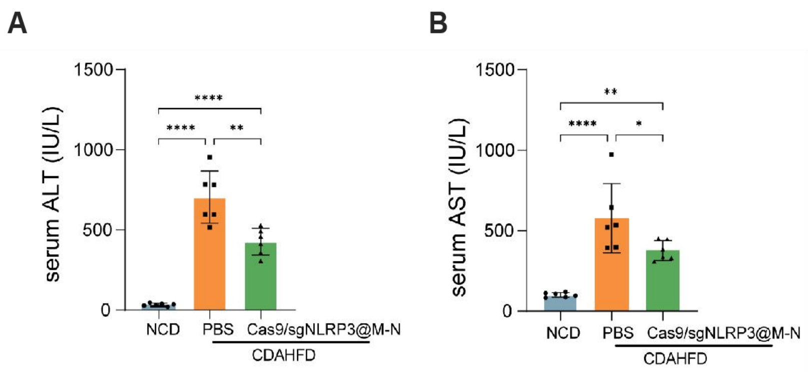

Figure S17. A) ALT and B) AST levels in serum of NCD, PBS, and Cas9/gNLRP3@M-N groups. n = 6 per group. Statistical significances were calculated via the one-way ANOVA; *$p < 0.05$, **$p < 0.01$ and ****$p < 0.0001$.

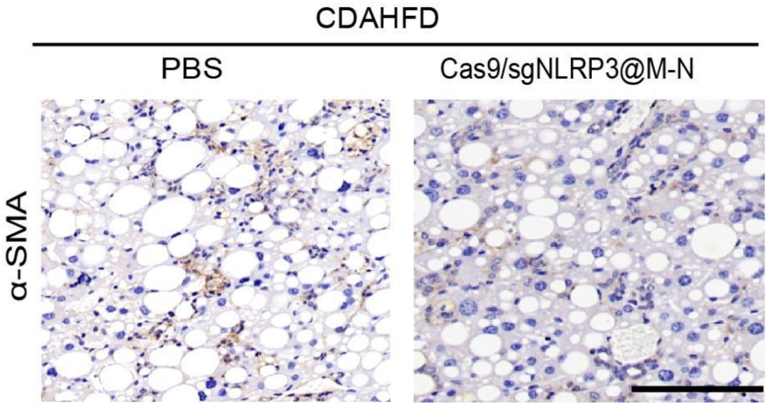

Figure S18. Representative images of α-SMA immunostaining in PBS and Cas9/gNLRP3@M-N groups. scale bar =100μm.

Supplementary Table 1. Sequences of primers used for real time RT-PCR.

Gene	Sense	Antisense
Fasn	CTGCGGAAACTTCAGGAAATG	GGTTCGGAATGCTATCCAGG
Cpt1α	AGGACCCTGAGGCATCTATT	ATGACCTCCTGGCATTCTCC
Pparα	TATTCGGCTGAAGCTGGTGTC	CTGGCATTTGTTCCGGTTCT
Srebp1c	GGCACTAAGTGCCCTCAACCT	GCCACATAGATCTCTGCCAGTGT
IL-18	TCCAACTGCAGACTGGCAC	CTGATGCTGGAGGTTGCAGA
IL-1	TCGTGAATGAGCAGACAG	ATCAGAGGCAAGGAGGAA
IL-6	TCCATCCAGTTGCCTTCTTG	GGTCTGTTGGGAGTGGTATC
Acta2	GTCCCAGACATCAGGGAGTAA	TCGGATACTTCAGCGTCAGGA
Timp1	CGAGACCACCTTATACCAGCG	ATGACTGGGGTGTAGGCGTA
Col1a1	GCTCCTCTTAGGGGCCACT	CCACGTCTCACCATTGGGG
GAPDH	GGTTGTCTCCTGCGACTTCA	TGGTCCAGGGTTTCTTACTCC

Supplementary Table 2. Sequences of DNA oligo.

Gene Names	Gene Names	sequences
Target sequences	NLRP3 gRNA-1	GACGAGTGTCCGTTGCAAGCTGG
	NLRP3 gRNA-2	AAGGACAGGAACGCGCGTCTAGG
	NLRP3 gRNA-3	GAAGATTACCCGCCCGAGAAAGG
Primers for sgRNA transcription template	NLRP3 gRNA-F1	5'TTAATACGACTCACTATAGGGGACGAGTGT CCGTTGCAAGCGTTTTAGAGCTAGAAATA -3'
	NLRP3 gRNA-F2	5'TTAATACGACTCACTATAGGGAAGGACAGG AACGCGCGTCTGTTTTAGAGCTAGAAATA -3'
	NLRP3 gRNA-F3	5'TTAATACGACTCACTATAGGGGAAGATTAC CCGCCCGAGAAGTTTTAGAGCTAGAAATA -3'
in vitro digestion PCR primer/T7 PCR primer	NLRP3 gRNA1,3-F	CCTGCACTGCCAGTGTGGACCTAAG
	NLRP3 gRNA1,3-R	GTTGGGAGCTTCAGTTGTGCAAGAT
	NLRP3 gRNA2-F	CACAACCATAGGCTTCAAAC
	NLRP3 gRNA2-R	TTGAAGAGCTTTCCCAGTGC
